# Humidity Control Strategies for Solid-State Fermentation: Capillary Water Supply by Water-Retention Materials and Negative-Pressure Auto-controlled Irrigation

**DOI:** 10.3389/fbioe.2019.00263

**Published:** 2019-10-17

**Authors:** Qin He, Huadong Peng, Mengyao Sheng, Shishan Hu, Jiguo Qiu, Jiayu Gu

**Affiliations:** ^1^Department of Microbiology, Key Lab of Microbiological Engineering of Agricultural Environment, Ministry of Agriculture, College of Life Sciences, Nanjing Agricultural University, Nanjing, China; ^2^Imperial College Centre for Synthetic Biology and Department of Bioengineering, Imperial College London, London, United Kingdom; ^3^Nantong Lianhai Weijing Biology Co., Ltd., Haimen, China

**Keywords:** capillary water, humidity control strategies, solid-state fermentation, negative-pressure auto-controlled irrigation, superabsorbent polymers

## Abstract

Solid-state fermentation (SSF) has regained interest owing to its advantages in solid waste treatment and fermentation industries. However, heterogeneous heat and mass transfer are often caused by the absence of free water and noticeable water loss from microbial utilization and moisture evaporation in SSF. It is necessary to explore more effective ways to solve issues of water loss and water supplement in SSF based on online capillary water monitoring, because capillary water is the dominant form of water that is present and lost in substrate. Two novel capillary-water supply strategies were proposed, established and evaluated using three selected reference strains, including water-retention materials and negative-pressure auto-controlled irrigation (NPACI). This study employed superabsorbent polymer, a kind of water-retention material to enhance enzyme productivity with the most significant increase of 2.47 times. Moreover, the combination of NPACI and 0.1% superabsorbent polymers increased productivity by 2.80-fold, together with lowered gradients of temperature, moisture and products. Furthermore, a modified liquid-supply SSF was constructed through successful capillary water control by proposed humidity control strategies. This modified SSF system could address the shortcomings of inhomogeneous culture of traditional SSF.

## Introduction

Presently, solid-state fermentation (SSF) has been widely used for the production of industrially relevant compounds such as organic acids, flavors, ethanol and enzymes (Aguilera-Carbo et al., 2009; He and Chen, 2013a), owing to its excellent features involving the process in absence or near absence of free water (Singhania et al., 2010). Most SSF systems could be regarded as a three-phase structure, i.e., continuous gas phase, low moisture phase and agro-industrial solid wastes with lower thermal conductivity. The characteristics of this three-phase structure result in a shortage of inhomogeneous heat and mass transfer and limited microbial growth in SSF (Krishna, 2005), leading to an increased localized temperature, and decreased moisture, microbial biomass and productivity.

Enough available water possessed during fermentation could alleviate this problem because water can dissolve and help transfer nutrients. Moreover, water evaporation could take heat away and it could remove up to 80% of the generated heat in many cases (Gutierrez-Rojas et al., 1996; Krishna, 2005). However, water evaporation, microbial consumption, etc., often cause obvious available water loss, especially in a forced aeration system (He and Chen, 2015). This may lead to uneven distribution of water in both horizontal and vertical directions, and eventually generates water stress (Lonsane et al., 1992). Thus, it is necessary to replenish the lost water caused by moisture evaporation and microbial utilization in SSF (Hölker and Lenz, 2005). However, adding too much water would compact solid-substrate, impede oxygen transfer, and favor contamination. On the other side, too little moisture would inhibit microbial growth and enzymes production, and limit nutritional transfer (He and Chen, 2013a). The solid substrate must possess appropriate available moisture to support microbial growth and metabolism. Therefore, matrix water supply should be controlled online to support microbial growth and metabolism and exclude adverse effects (Gervais and Molin, 2003).

Theoretically, moisture status could be divided into hygroscopic water, capillary water and free water based on the moisture characteristics of the porous substrate (He and Chen, 2015). Water molecules that can be absorbed by substrate from the atmosphere through surface forces make up hygroscopic water. The hygroscopic water content is subject to the moisture variation, which could not be used by microorganism because hygroscopic water was firmly bound to the substrate. Capillary water is located in the capillary space and is available to and could be consumed directly by microorganisms. Capillary water was proved to be the major water present and lost in SSF (He and Chen, 2015). Free water, also called gravity water, is the excess water located in the aeration-pore space. Water saturated substrate affords small aeration space. Thus, this excess water is always unfavorable for microbial growth and could not be supplied in most aerobic SSF. Therefore, the online control of capillary water supply is the most effective way to solve the problem of substrate dehydration in SSF.

The non-pollution water-retention materials (WRMs), especially superabsorbent polymers (SAPs), could not only absorb water many hundred times their dry weight, but also exhibit excellent characteristics of water retention and supply. WRMs are excellent water-managing materials for agricultural purposes in drought-prone or even desert areas (Wan et al., 2008). The essentiality of WRMs in improving physical features of soil, such as porosity and water-holding capacity, has been confirmed (Yang et al., 2014), which provides a possibility to test the feasibility of water supply in SSF.

The capillary water movement was decided by the matrix-water potential (MWP) in SSF from regions of higher water potential to regions of lower water potential. So, it is reasonable to suggest another novel method for capillary water supply, based on water potential controlled by energy state, namely the negative-pressure auto-controlled irrigation (NPACI) technology.

This study constructed two available water supply strategies based on WRMs and NPACI. Three referenced strains characterized by different fermentation periods and water requirements were selected to evaluate their fermentation efficiency in cellulase, spores and laccase production, respectively. The key factors of water supplying in SSF were determined and compared between these three strains. The purpose of this study is to propose a successful capillary water control strategy, guiding online control of available water for industrialized SSF.

## Materials and Methods

### Water-Retention Materials and Its Physical Properties

Water-retention materials (WRMs) include superabsorbent polymers (SAPs), polyurethane foam (PUF) and non-woven fabrics, etc. Two kinds of SAPs, AQUASORB 3005 KL (particle size: 1~3.6 mm) and 3,005 KM gels (size: 0.3~0.7 mm) were purchased from SNF Company, France. PUF was treated as previously described (He and Chen, 2013b). Non-woven fabrics and sand were bought in a local market. Water absorbency is the amount of water absorbed by SAPs (in g per g of dried particle) (Raju et al., 2002). Water evaporation is described by the moisture-release curve of water-saturated SAPs (in g per g of dried particle).

### Steam-Exploded Corn Stover (SECS) Preparation and Its Physical Properties

Corn stover (CS) was harvested in suburban Beijing, China. It was air-dried, smashed and steam-exploded at 1.5 MPa for 5 min (Chen et al., 2011). Steam-exploded corn stover (SECS) was then washed with 60°C water and air-dried for detoxification (Li and Chen, 2008). SECS were prepared with two different particle sizes (0.43 and 5 mm) before sterilization. The sterilized medium was cultured without inoculation at 30°C to study the simulated evaporation. We sampled every 24 h and dried at 105°C for weighing. Evaporation rate (ER, g^**.**^g^−1^.*h*^−1^) was calculated:

(1) $$\begin{array}{r} ER=\frac{m_{j}-m_{i}}{m_{s}\times \left(t_{j}-t_{i}\right)} \end{array}$$

where *m*_*j*_ and *m*_*i*_, are the matrix mass at the time of *j* and *i* (*j*>*i*), respectively, g; *m*_*s*_, dry mass of substrate, g.

### Measurement of Substrate-Water Characteristic Curve

The curve showed the relations between matrix-water potential (MWP) and moisture content (MC), and was fitted using Van Genuchten (1980) model by Retc software (Zhao et al., 2007),

(2) $$\begin{array}{r} \theta =\theta r+\frac{\theta s-\theta r}{{\left[1+{\left(\alpha \times h\right)}^{n}\right]}^{m}} \end{array}$$

where θ, θ*r*, and θ*s* were the matrix, hygroscopic and saturated water content, respectively, %; α and *n* were the fitting parameters, non-dimensional; *m* = 1–1/*n*.

MWP was measured using a modified centrifugal method (Jauregui et al., 1981). The calculation formula of water potential was as follows:

(3) $$\begin{array}{r} H=h\left(r_{1}-\frac{h}{2}\right)\times \frac{w^{2}}{g}=h\left(r_{1}-\frac{h}{2}\right)\times 1.118\times 10^{-5}n^{2} \end{array}$$

Where *H* was the water potential, mm; *n* was the rotating speed, rpm; *r*_1_ was the rotor radius, mm; *h* was the center of gravity height, mm.

### Reference Strains, Culture Medium, and Product Measurement

*Trichoderma reesei* YG3, *Trichoderma harzianum* 3.5364, and *Fomes fomentarius* 5.132 were selected as reference strains (Table S1). For *T. reesei*, its solid-state medium (SSM) and cellulase activity measurement were described previously (Chen et al., 2011). At the end of SSF, the culture was weighed and divided into two equal parts. One half was dried at 60°C to measure the MC and dry weight. The other half culture was added first to 0.02 M citrate buffer (pH 4.8) (solid-liquid ratio of 1:20), then shaken at 150 rpm for 2 h and centrifuged at 4,000 rpm for 10 min. The obtained supernatant was used to hydrolyze Whatman No. 1 filter paper at 50°C for 60 min to test the glucose produced according to Ghose's method (Ghose, 1987). Cellulase activity (filter paper activity was tested in this paper) was expressed as international units, defined as the amount of enzyme required to liberate 1 μmol glucose per minute under the assay conditions and reported on the basis of per gram of dry substrate. All experiments were carried out in triplicate. Biomass estimation was measured by a modified method using nucleic acid as a biomass indicator (Wei et al., 2006). The temperature and humidity were recorded every 2 cm bed depth at intervals by Pt100 and humidity sensors after calibration.

SSM for *T. harzianum* consisted of 4 g SECS, 0.4 g glucose, 0.4 g peptone, 0.4 g NH_4_NO_3_, 0.04 g KH_2_PO_4_, and 0.2 g MgSO_4_ with 16 ml water, pH 5.5. After sterilization, five microbial pieces made by a hole punch (each 0.5 cm in diameter) from leading edge of mycelia on PDA plate were inoculated into a 200 ml tissue culture bottle and incubated at 28°C. The number of spores was measured using methods reported previously (Chen and He, 2013).

For *F. fomentarius*, SSM consisted of 4 g SECS, 0.32 g glucose, 0.32 g CuSO_4_, 0.128 g (NH_4_)_2_SO_4_, 0.0064 g MgSO_4_, 0.0064 g MnSO_4_, and 0.0064 g NaCl with 16 ml 25% wheat bran extract, pH 5.0. The inoculation and culture were similar to *T. harzianum* except that they were incubated at 30 days under 30°C. The activity was defined as the amount of laccase required to oxidize 1 μmol substrate per min at 25°C and measured as previously reported (Qiu and Chen, 2008).

### Capillary Water Supplied by SAPs in Liquid-Supply SSF

SAPs were firstly equilibrated in deionized water. Swollen gel and SSM were put into a tissue culture bottle (2.5 cm × 10 cm) with a packing depth of 6 cm and wet density of 400 kg/m^3^ before sterilization at 121°C for 35 min. After that, the strains were cultured in their optimal MC according to the preliminary test. Effect of SAPs types, as well as SAPs adding amount and matrix particle size, were further carried out by response surface experiments (Table S2).

### Construction of Negative-Pressure Auto-controlled Irrigation (NPACI)

**Figure 1:**
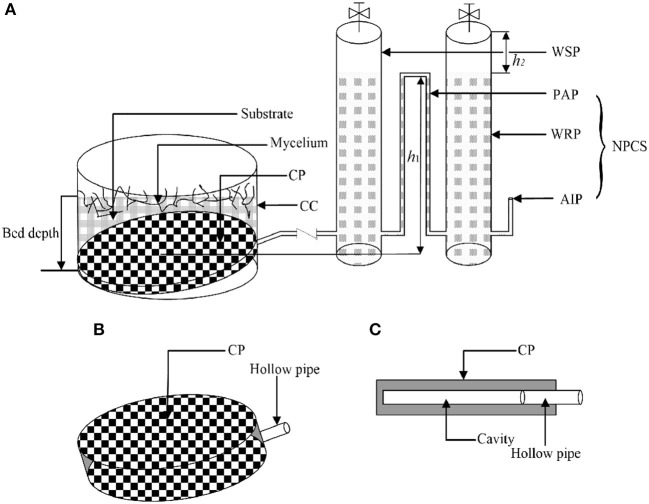
Negative-pressure auto-controlled irrigation device (NPACI; Islam et al., 2011). **(A)** Sketch map of NPACI. CC, closed culture chamber; CP, ceramic plate; WSP, water supply pipe; NPCS, negative pressure controlling system; AIP, air inlet pipe; PCP, pressure-controlling pipe; WRP, water-reserving pipe. **(B)** Magnifying map of CP. **(C)** Profile drawing of CP.

Negative-pressure auto-controlled irrigation (NPACI) (Figure 1; Islam et al., 2011) is mainly composed of a closed culture chamber (CC), a water-supplying micro-porous ceramic plate (CP), a water supply pipe (WSP), and a negative-pressure controlling system (NPCS; Islam et al., 2011). CP and NPCS are the most important elements. CP is made from a novel ceramic material which allows only water molecules to seep through and denies air molecules. NPCS mainly contains an air inlet pipe (AIP), a pressure-controlling pipe (PCP) and a water-reserving pipe (WRP). WSP is connected to CP, which is installed at the bottom of CC. WSP is located higher than the top of CP, thus water could infiltrate slowly from CP into CC. Because of gravity, the water pressure in the cavity of CP reduces with increasing height of PCP and remains constant at a particular height. The different height of PCP, which could be considered as water supply tension, can create a particular pressure potential in the cavity of CP. During fermentation, substrate water is utilized by strains, and water potential is then formed with the decreased MC. The water inside of CP is slowly penetrating the substrate due to the effect of MPW, which makes the water in WSP enter into CP. This would enable moisture in WRP go through PCP into WSP under the atmosphere pressure. In this way, air pressure balance will be reached between WSP and AIP. Consequently, water is continuously transported into the substrate layer of the device by the negative pressure caused by water consumption. The negative pressure is equal to the outlet pressure of WSP (–ρ*gh*_1_), where *h*_1_ is the height of PCP, m; ρ is the water density, kg/m^3^. In this way, different MC can be monitored by adjusting the height of PCP. The reference strains were cultured in the pressure potential equivalent to their optimal MC according to the preliminary test and the substrate-water characteristic curve.

### Capillary Water Supplied by NPACI in Liquid-Supply SSF

Procedures of capillary water supply are listed as follows. (1) Sterilized SECS is put between the downside of CP and bottom of CC to form a sloping surface; (2) two layers of sterile gauze are used to enclose the CP and put the CP diagonally, sterilized substrate is stacked upside, and then CC is closed; (3) WSP and WRP are fixed, and the water-level gauge is installed; (4) PCP is sealed, and its height is adjusted to satisfy the required negative water potential; (5) the hollow pipe of CP is connected with WSP; (6) the valve is closed between CP and WSP, AIP, and the upper valve of WSP is closed, PCP is clamped. Then the valve of PCP is opened to fill the pipe with sterile water, then the valve is closed; (7) similar procedure as (6), WRP is filled with sterile water; (8) the clip is removed, the AIP is plugged on, and the valve between CP and WSP is opened, water enters the device automatically.

## Results

### Proposed Water Supply Strategy on Water Distribution and Evaporation Analysis

Although gaseous water flows exist under arid conditions, liquid water contributes most to heat and mass transfer in SSF because the transportation of liquid water is the main water movement pattern in the solid matrix. It is essential to clarify the mechanism of water movement and loss before proposing water supply strategies. Water distribution, transportation, and loss is not only driven by capillary force, but also by a variety of other forces, such as gravity, Darcy's resistance, phase change, and interaction between the gaseous mixture and liquid water, inertial force caused by liquid film movement and liquid gravity (Richard et al., 2004; Chen, 2013; Zhang et al., 2017).

**Figure 2:**
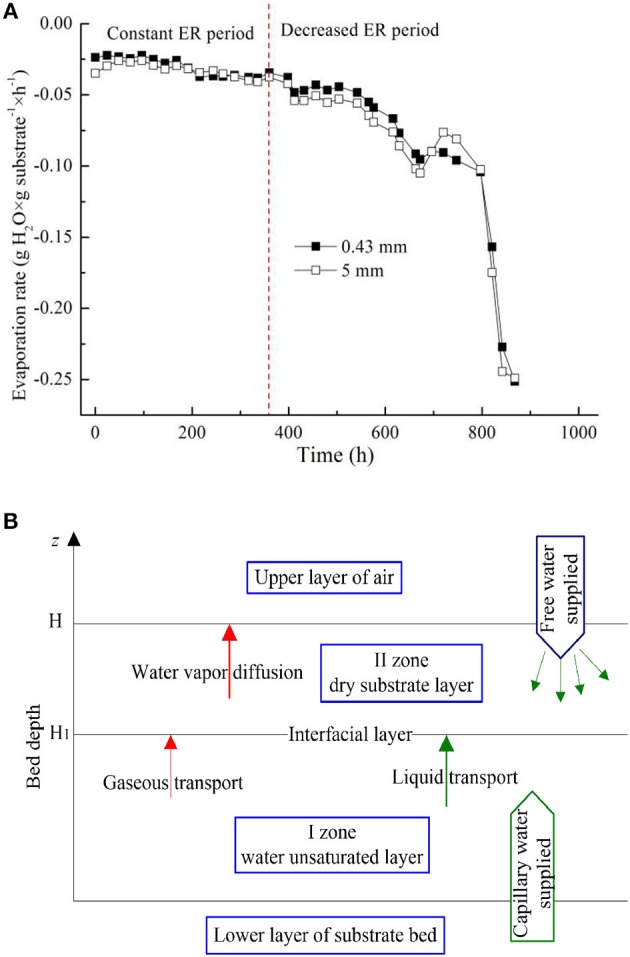
Vertical moisture stratification of the substrate according to simulated water evaporation. **(A)** Water evaporation of the substrate; **(B)** schema of vertical moisture stratification of the substrate.

Considering that water evaporation always makes the greatest contribution to heat transfer and water loss, simulated evaporation process (Figure 2A) using sterilized medium only was studied. At the beginning (0–15 days), the absorbed water on the matrix surface evaporated first. The surface evaporation happened at a constant evaporation rate (ER), then the humidity gradient occurred between the exterior surface and inner surface of substrate. This gradient promoted liquid water movement and evaporation from the interior to the surface of matrix. However, the internal liquid water moving rate was lower than the surface water evaporation rate. Thus, the substrate surface began to dry after a period of surface evaporation, and the internal liquid water began to evaporate. The evaporation entered a period of decreased ER, during which ER mainly depended on the water vapor partial pressure (*P*_w_) at an evaporating meniscus in small capillary tubes. According to Kelvin's law (Saguy et al., 2005), *P*_w_ at the capillary meniscus is proportional to the capillary tube radius. Lower radius means lower *P*_w_, which results in less mass transfer impetus and decreased ER.

In actual SSF process, the solid substrate could be considered as typical water unsaturated porous media. The surface moisture would be exhausted when water was seriously lost or used by strains and ventilation, and then pore water was used. A dry substrate layer (total water content about 10%) formed when the absorbed water was utterly lost. After the emergence of the dry layer, the evaporation boundary is no longer on the surface of the substrate, but in the interior. The rate of evaporation reduced significantly. The rate of water loss mainly depends on the vapor diffusion, and is also related to the dry layer thickness. This phenomenon is called vertical moisture stratification (MS) of substrate (Figure 2B). The effect of MS on internal heat and moisture migration in substrate bed should be mentioned when studying the actual SSF system. This phenomenon also explained the importance of packing depth to SSF. Additionally, according to the intrinsic characteristics of MS of substrate, water control strategy is suggested: free water should be supplied above and capillary water supplied at the bottom of the substrate in SSF (Figure 2B). This study tried to achieve capillary water supplement based on the proposed principle.

### Capillary Water Supplied by Water-Retention Materials (WRMs) in Liquid-Supply SSF

Materials with excellent water-holding capacities could offer an opportunity for moisture supplement under capillary force. Superabsorbent polymers (SAPs) are superior materials referring to water-insoluble hydrophilic polymers, which can swell and absorb water, saline solutions or physiological fluids as high as 10–1,000 times their own weight (Wan et al., 2008; Gawande and Mungray, 2015). Its morphology changed much after water absorption (Figure S1). Both the two SAPs exhibited functional water retention capacity, and they could quickly achieve water saturation in 1 h (Figure S2a). Water absorption value of AQUASORB 3005 KL and KM gels reached 95.05 and 144.82 within 1 h, respectively. KM had a stronger water-absorbing ability, 1.52 times of KL. Water surface evaporation of SAPs samples was wholly finished at about 15 days (Figure S2b). In comparison, polyurethane foam (PUF), porous material as the popular hydrophilic substrate, could only retain water in 5 days. For non-woven fabrics, sand and other materials, their water retention performance was only about 1 day, which was significantly inferior to SAPs (data not shown).

Among these three reference strains, both *T. reesei* and *F. fomentarius* produce hydrolases but the latter employs a longer fermentation period (5–7 days vs. 28–30 days). *T. harzianum* was chosen because viable spores are another kind of important products produced by SSF. In addition, its fermentation period (10–12 days) was between *T. reesei* and *F. fomentarius*.

**Figure 3:**
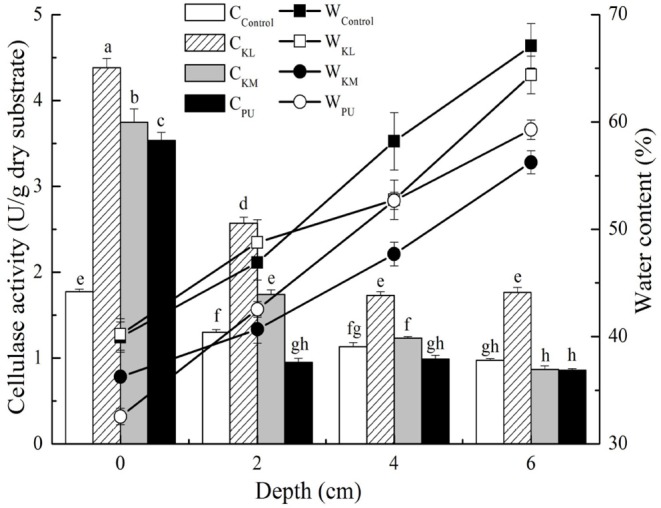
Effect of WRMs types on cellulase production. Bars represent standard errors and different letters above the histogram indicate significant differences (*P* < 0.05) according to Tukey test.

**Figure 4:**
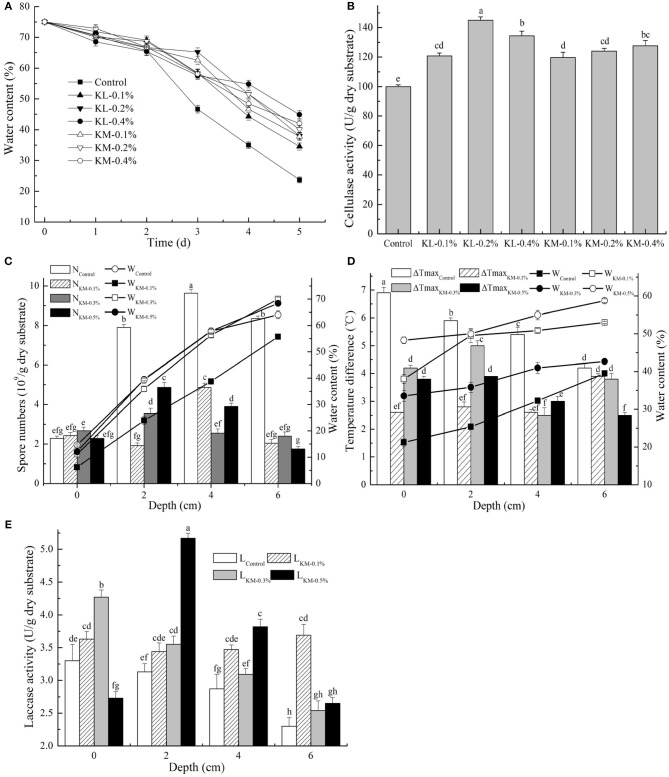
Effect of SAPs amount on three model strains fermentation. **(A)** MC and **(B)** enzyme activity at the packing depth of 4 cm in cellulase production; **(C)** variance in MC and spore number under different bed depths after *T. harzianum* fermentation; **(D)** maximum temperature difference and MC; and **(E)** laccase activities under different bed depth after laccase fermentation. Bars represent standard errors and different letters above the histogram indicate significant differences (*P* < 0.05) according to Tukey test.

Effects of different water-retention materials (WRMs) were evaluated first on steam-exploded corn stover (SECS) water-holding capacity and cellulase production at different packing depths in inoculated SSF (hot model) or not (cold model). First it revealed better water retention capacity in SSF than cold model culture owing to the microbial coverage (Figure S3). The lowest residual MC was 26.77 vs. 14.23% after fermentation. SAPs realized better water holding effects, and the final MC reached 35.82 and 37.90% in KL and KM test at the top substrate. It increased by 33.81 and 41.58% compared with the control test of hot model, respectively. Besides, compared with the control test, SAPs addition could effectively minimize the humidity gradient at any given bed depths, although moisture gradient still existed. PUF could hold some water and provide low humidity gradient, but the total MC was lower than SAPs-treated samples after fermentation. Thus, SAPs behave better water retention capacity and are appropriate water-supply carrier for SSF. SAPs dramatically increased the produced enzyme activity. The increment was as much as 2-fold of other tests (Figure 3) (regarding intrinsic characteristic of SSF process, microbial gradients were generated within the culture). It peaked out at 2.47 times at the top position in control group. MC and enzyme activity varied with SAPs amounts (Figures 4A,B) although significant positive effect was reached by SAPs addition. The larger amount of added SAPs, the smaller decrement of MC after fermentation. The optimal activity was achieved when KL adding amount was 0.2%, which was enhanced by 45.01% compared with control.

Effect of SAPs amount on *T. harzianum* production (Figure 4C) revealed that the water holding capacity did not improve accordingly with the increasing SAPs amount. It clearly showed that SAPs could efficiently control the humidity, and further reduce water gradient and promote heat transfer during fermentation. It was beneficial to maintain the optimum fermentation temperature and aid microbial growth and metabolism. However, through the efficiently controlled temperature and water, the spore production capacity did not increase but reduce. The largest biomass value was found in the control test, reaching 4.77 times of the lowest in a corresponding fermentation site.

Added SAPs amount affected laccase production by a white-rot fungus differently in temperature, MC, and enzyme activity (Figures 4D,E). During fermentation, the maximum temperature difference in SAPs-treated group was smaller than that in the control group. A smaller temperature gradient below 0.4°C/cm predicted a better heat transfer efficiency. In the control test, the highest temperature was 6.9°C higher than the initial temperature. When referring to MC, it exhibited apparent differences in absolute MC at the same loading depth as well as humidity gradients at different packing levels. SAPs provided excellent water retention capacity in enzyme production, especially hydrolase. The highest enzyme activity was found at the SAPs adding dose of 0.5%. However, the economic amount was selected as 0.1% based on the enzyme activity gradient.

SAPs particle had a positive effect on cellulase activity (Figures S4a–d). It was also a significant factor in conidial production by *T. harzianum* (Figures S4e–h), larger SAPs were beneficial to SSF. Larger particles increased the porosity and ventilation of the fermentation system, which was helpful for sporulation at the late fermentation stage. In Figures S4i–l, it can be seen that laccase production was different from cellulase although both of them were hydrolases. The particle size of SAPs had a significant effect on laccase formation. This difference existed because it needed a long fermentation period in laccase production, small particles of SAPs behaved better performance of water retention than large particles at the late fermentation stage. Thus, water supply was more affected by particle size of SAPs.

### Capillary Water Supplied by Negative-Pressure Auto-controlled Irrigation (NPACI) in Liquid-Supply SSF

**Figure 5:**
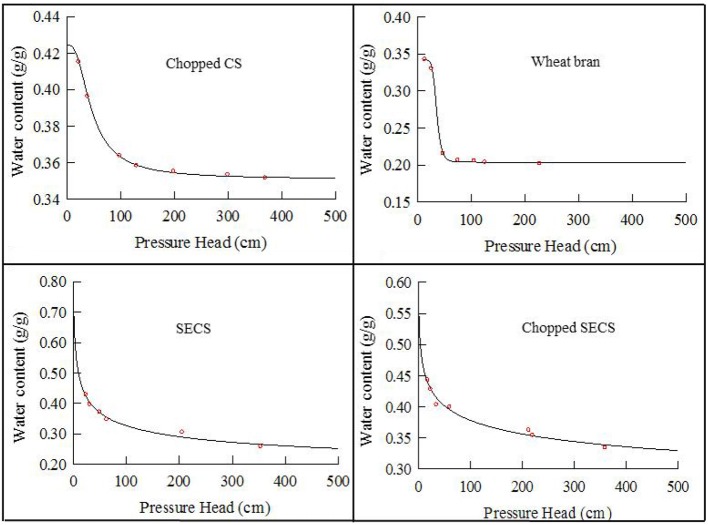
Fitting water characteristic curve of different substrates by RETC software.

The substrate-water characteristic curve showed the relationships between surface adsorption capacity of matrix and capillary force of matrix pores acting on the water in water-unsaturated substrate (Figure 5; Van Genuchten, 1980). *R*^2^ value of the fitted curve reached 0.97 or higher (Table S3). Steam-exploded samples had higher saturated MC than wheat bran and CS because the porosity increased after steam explosion. Higher porosity is beneficial to higher water retention and forced aeration. The residual MC, i.e., hygroscopic water in chopped CS and wheat bran, was both higher, which led to lower available water in these two substrates. Compared with the smashing samples, raw SECS was more suitable for SSF considering the water characteristic parameters as well as the energy required in the grinding process.

Based on the energy status and characteristics of substrate moisture, we proposed a way to use the negative water potential to achieve the online supply and quantitatively control of capillary water. We constructed the negative-pressure auto-controlled irrigation (NPACI) device (Figure 1) and technology, and it could provide a highly accurate continuous flow of water in SSF with a required moisture status maintained. For example, when the strain cultivated in its required MC of 75%, the reported final MC was <55% in both static SSF and gas double-dynamic SSF (He and Chen, 2015). Matrix-water potential (MWP) corresponding to a constant MC of 75% was controlled online in this study. Meanwhile, SAPs were additionally added to study the combined effects of SAPs and NPACI (Figure 1).

**Figure 6:**
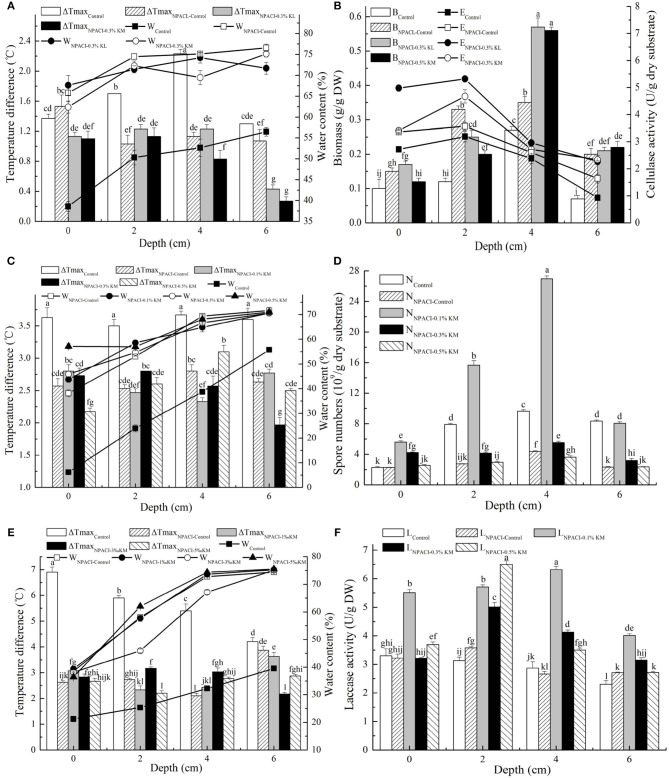
Parameters difference in three model strains fermentation by NPACI controlling. **(A,C,E)** Maximum temperature difference and MC under different bed depths in three model strains fermentation; **(B)** biomass and enzyme activities under different bed depths after cellulase production; **(D)** spore number under bed depths after *T. harzianum* culture; **(F)** laccase activity under bed depths after fermentation. Bars represent standard errors and different letters above the histogram indicate significant differences (*P* < 0.05) according to Tukey test.

Results showed the lower temperature improvement and smoother temperature gradient in NPACI group than that in control group (no water supplied), which was related to the supply of sufficient water (Figure 6A). When SAPs were added, the temperature heterogeneity in both fermentation period and packing depth further reduced, especially the maximum temperature variation at the bottom of the substrate. In Figure 6A, NPACI succeeded in maintaining substrate moisture after fermentation in its controlled group. MC did not change with the packing depths, while its variation was depended on the gravitational potential energy of the substrate water. On the contrary, MC in the control group changed considerably, indicating that there was a significant humidity gradient.

The biomass of the control group produced at different depths was significantly lower than that of NPACI treated at the late fermentation stage. NPACI had a noticeable promotion effect with the nearly doubled biomass (Figure 6B). In addition, biomass increased further in SAPs-added group, which was 3–4 times of that in control group, and it reached the highest value at a depth of 4 cm. This phenomenon proved that SAPs had a further significant effect on biomass promotion on the premise of controlled water.

Cellulase activity obtained from the control group was the lowest, with a significant gradient existed (Figure 6B). Activity at the top substrate was about 3 times that at the packing depth of 6 cm. The highest activity was found at 2 cm, 3.43 times the lowest. For NPACI-control group, the total enzyme activity increased with a lower gradient than that of the control group, and the highest activity was 2.18 times the lowest. For NPACI with SAPs-added, enzyme activity was much higher than that of control group. The highest activity was 2.44 and 1.49 times of control test and NPACI-control test, respectively.

The maximum temperature difference was studied at different stacking depths during *T. harzianum* culture (Figure 6C). NPACI had a noticeable effect on decreasing temperature rise. In SAPs-added group, both the lowest temperature rise and the lowest temperature gradient under stacking depth were found at an adding amount of 0.1%. NPACI controlled MC well-compared with the control group during fermentation, and the humidity gradient was reduced after fermentation (Figure 6C). In the NPACI groups with different amounts of SAPs, MC was further maintained and humidity gradient was well-controlled. For *T. harzianum*, spore was the final product. The spore amount decreased in most NPACI experiments (Figure 6D). However, in the group of NPACI with 0.1% SAPs, the biomass increased obviously, which reached 2.80 times at a depth of 4 cm.

The temperature variation decreased significantly in laccase production by *F. fomentarius* using NPACI (Figure 6E), especially at a stacking depth of <4 cm. The temperature change was 2.65 times as much as that obtained at the top of substrate in NPACI group. The highest temperature difference reached 1.8°C in NPACI group vs. 2.7°C in control group. NPACI not only reduced the temperature difference during the fermentation period, supplying a micro-environment of nearly constant temperature, but also offered a lower temperature gradient. In the group of NPACI with SAPs, the temperature difference also decreased. Also, NPACI can better control MC and humidity gradient under the stacking depth during fermentation (Figure 6E). When compared with the control group, the activity of NPACI group was higher and more uniform (Figure 6F). The combination of NPACI and SAPs improved enzyme activity, especially with 0.1% SAPs. Besides, the activity gradient was not significantly increased compared with that of control group. Hence, it was more suitable for laccase production. Although the highest activity appeared in the group with 0.5% SAPs, it was not suitable for practical application because of the higher enzyme activity gradient (the highest activity was 2.4 times of the lowest activity) and more SAPs required.

## Discussion

The lost capillary water in SSF was supplemented with NPACI or SAPs or both. SAPs significantly increased enzyme activity. During fermentation, the stored water and nutrients were released from SAPs slowly as required by microbes to improve microbial growth under water limited-conditions (Zhao et al., 2007). Higher biomass secreted more enzyme protein and thus resulted in higher enzyme activities. Beyond that, the positive performance could relate to the increased porosity and aeration from large particles of SAPs. Higher porosity resulted in better ventilation and heat dissipation. Several studies have proved the importance of SAPs in improving physical properties of soil such as porosity, structure and water-holding capacity (Kabiri et al., 2003; Islam et al., 2011).

The requirements of retaining water were also proved to be different in varied microbes and fermentation products. The negative effect was observed in *T. harzianum*, because lower MC was combined with the best sporulation conditions (Reynaldo et al., 2015) which required lower water stress. Fungal growth was vigorous under the initial MC firstly. Besides, SAPs addition would increase the ventilation effect, which further increased the biomass. Then, the active mycelium yielded increased spores after entering the sporulation stage. However, more SAPs addition did not produce more spores, because the provided higher MC made substrate wet, making it non-conducive to spores formation. Furthermore, wet fungus would induce the formation of water film on the substrate surface and leave limited ventilation. Therefore, spore production was difficult. This agrees with the fact that a large number of wet mycelium formed at the late stage of fermentation, but with a low spore yield (Reynaldo et al., 2015).

Regarding the inhomogeneity characteristics of SSF, a heterogeneous environment was required by fungal metabolism (Lopez-Perez et al., 2015). Thus, microbial gradients were inevitably produced (biomass, nutrients, enzyme, pH, etc.). Additionally, substrates used in SSF always presented a low thermal conductivity, resulting in differences of heat accumulation (Pandey, 2003). In fact, not only the reactor type influences heat transfer differently (e.g., tray bioreactor vs. agitated or aerated bioreactor), but also the bed height and type of internal air circulation do (Figueroa-Montero et al., 2011). It was (Dorta and Arcas, 1998) stated that thermal and humidity gradients generated when scaling up, and the gradients had an unfavorable effect on growth. Porosity adjustment was suggested in order to avoid drastic water loss. Water, the solvent for mass and heat transfer due to its satisfied transfer coefficient, could solve the aforementioned problems. When SAPs were incorporated with porous media, it was presumed that they retained large quantities of water and nutrients, which were released as required by microbes. Thus, microbial growth could be improved by controlled water supply. In the following fermentation stage, enzyme secretion process needed more water whereas sporulation did not, so both the hydrolases were enhanced in liquid-supply SSF, but the spores did not increase, and the differences could be related to the physical properties and water characteristics of the solid substrate. Matrix holding different water content showed varied water characteristics in our previous study by a low field nuclear magnetic resonance (He and Chen, 2015). Adding different amounts of SAP arouse content changes in available water which affects the microbial fermentation.

Although SAPs can provide capillary water for SSF, there were still shortages that the quantity of supplied capillary water was independent from in the fermentation process, and it only provided limited water at late stage of a long fermentation period. This technology is required to be further improved based on the loss of available water. However, it was difficult to measure the produced metabolic water by aerobes (Hölker and Lenz, 2005). In general, water flow toward microbial activity sites along free energy gradients in SSF. Moreover, the tendency is toward energy equilibrium in any system. Thus, MWP is an important parameter for characterizing substrate-water energy status, with the potential energy of water almost always negative. NPACI technology was adopted to solve this problem based on MWP theory. Decreased MC difference and smaller water gradient, and temperature were well-controlled in enzyme production, so fermentation product yield increased. When using both NPACI with SAPs, productivity was further improved because of better ventilation.

Previous studies proved that water supply could control MC, provide a suitable microenvironment for microbial growth and metabolism, and effectively improve fermentation yield (Table 1). For example, cellulase activity increased by 33.6% (Ito et al., 2011), butanol production increased by 47% (He and Chen, 2013b). Reported moisture control strategies in SSF included: culture using a higher initial moisture or inlet air of high humidity (96–98%) to maintain MC (Gervais and Molin, 2003) or by water spraying (Lu et al., 2003), or water supplied by wet polymers (Ito et al., 2011), or even directly drop of distilled water (Torrado et al., 2011). For example, Dey and Banerjee (2012) utilized an improved tray bioreactor: a tray was placed inside a vessel, and liquid was placed between the container and tray, the sterile compressed air carried moisture into the substrate to maintain its moisture. The present water supply methods used in the solid-state fermentation process, including spraying, drip irrigation, etc. Most of them are free water supply based on positive pressure. Substrates are easy to reach saturation after water supply and dried after a period of culture because of both evaporation and microbial utilization. This will result in dry and wet alternates in a specific substrate region, and may cause flooding and nutrient waste and even lead to bacterial infection.

**Table 1:** Comparison of water supply methods.

**Fermentor type**	**Replenished method**	**Decreased water loss method**	**Benefits**	**References**
SAPs-Static SSF	SAPs supplying		Highest activity reached 2.47 times of the lowest	This study
NPACI- SAPs-SSF	NPACI combined with 0.1% SAPs		Highest activity reached 2.80 times of the lowest	This study
130-L Zymotis packed bed	Forced aeration with humidified air	Water jacket, heat exchanger plates, and forced aeration	Higher substrate loading, increase in cellulase productivity was 1.46 times	Roussos et al., 1993
Erlenmeyer flask	Water added directly		An increase of 17% in citric acid	Torrado et al., 2011
Pilot static bioreactor with periodic agitation	Water and steam addition	Cool air inlet and agitation	No significant deviations between set and measured humidity	Fernández et al., 1996
Tray in a vessel with a lower vessel downside to supply compressed humidity air	Inlet airlift the liquid into the upper vessel and touch the substrate bed	Compressed air	High α-amylase activity of 22,316 U/g substrate with reduced culture period	Dey and Banerjee, 2012
Pilot-scale wooden tray	Maintain air humidity of 90% by spraying cooled steam water	Turn over the medium and ventilation	Increased xylanase activity from 650 to more than 1,000 IU/g (dry koji)	Lu et al., 2003
0.72 m^3^ upright multi-tray conidiation chamber	Clean mist input from a moisture generator	Air conditioning, the bottom of each tray installed open mesh	Nearly saturated humidity maintained with a mean yield of 2.4 × 10^12^ conidia kg^−1^ rice	Ye et al., 2006
A lab-scale system consisting of 16 columns placed in a water bath	An airflow rate of 20 mL/min and an inlet air humidity of 80%	Forced aeration	44% increase in xylanase activity	Pirota et al., 2013
1.5-L scraped drum reactor and 35 L horizontal paddle mixer	Calculate water loss by measurement and model estimate water using on-line predict		A validated model accurately predicted MC	Nagel et al., 2001
Intermittently mixed, forcefully aerated SSF bioreactors	Manipulate inlet air at 100% humidity, drop outlet air humidity based on computer simulation	Intermittently mixed and forced aeration		von Meien et al., 2004; Khanahmadi et al., 2006
Non-airflow box with a moisture permeable fluoropolymer membrane	Moisture permeable fluoropolymer membrane		consistent temperature and MC; the largest increase of secreted protein and enzyme activity were 20 and 65.7%, respectively	Ito et al., 2011, 2013
SSF using polyurethane foam (PUF) as an inert support	Free water supplied by PUF as an inert carrier		Increased amylase and nuclease P1 activity and short fermentation time	Zhu et al., 1994
Absorbent fermentation	Free water supplied by alkali-treated SECS		Acetone-Butanol-Ethanol concentration increased by 47%	He and Chen, 2013b

In order to avoid excessive fluctuations of matrix moisture, the amount of supplied water and appropriate adding time need to be calculated, predicted and controlled by detecting microbial physiological parameters, matrix moisture parameters, etc. From 1980s, with gradually developing interest in numerical simulation of heat and mass transfer in SSF, people did some research on liquid supply: define the initial MC by parametric optimization tools and algorithm (Thomas et al., 2013) or supply water based on water loss calculated by constructed model equations (Nagel et al., 2001; Khanahmadi et al., 2006). These methods provided a sufficient supply of moisture to the microorganisms. However, the operation is poorly implemented and wastes a lot of experimental results to achieve it. Therefore, the constructed models were often empirical and very few tests were conducted to investigate the variances in different fermentation stages. Thus, using this method is especially challenging to achieve online control on the fermentation stages and solve the problem of long-term water-supply needed in the long-period microorganism culture.

In conclusion, there were two strategies available to prevent water loss. Selected substrates, such as impregnated hemp (Weber et al., 1999), should have an unobvious decrease in water activity, even though it experienced massive changes in MC considering the shear damage to fungal hyphae. However, the substrate was often complicated because it contained various solid wastes with different water-retention features. Therefore, water must be added into the bed during fermentation to avoid water limitation. In detail, in laboratory scale, water was usually controlled by placing the bioreactor in a culture chamber with moist air saturated by saline solutions. At a larger scale, bioreactor was usually force aerated with water-saturated air (Bellon-Maurel et al., 2003). The humidification of air and recirculation of cooled exhaust gases enhanced MC control. However, in many cases, even if saturated air was supplied inlet, it was impossible to add water uniformly to a static bed. Furthermore, the transfer of heat from solid to the air in the bed increased its water-carrying capacity, and therefore water evaporated from the substrate (von Meien et al., 2004). Another strategy proposed was to calculate how much water must be replenished. These parameters can be obtained experimentally (Fernández et al., 1996), but it was expensive and time-consuming in large-scale SSF. Alternatively, reasonable estimates of the parameters can be predicted and obtained by computer simulation. This required a reliable mathematical model to describe the changes in temperature and MC. Inevitably, the relations among temperature, humidity, microbial growth, bed heterogeneity, etc., must be considered in the predicted model (von Meien et al., 2004).

Although the quantitative comparison was only a reference data considering varied strains, substrates, packing densities, depths, and substrate sizes, the humidity control strategies we proposed showed a definite advantage on increasing production, higher than those reported (Table 1). Among these strategies, the designed fermenter “Zymotis” presented an increase of 1.46 times in cellulase activity (Roussos et al., 1993). In contrast, the proposed SAPs strategy could enhance cellulase activity with the largest increase of 2.47 times, and NPACI combined with 0.1% SAPs increased 2.80 times in spore production of *T. harzianum*. Besides, temperature, moisture, microbial, and product gradients were all lowered with promoted uniform culture.

Industrialization of SSF, as a green technology of water and energy-saving, is meaningful to the social economy and environment. This study guides to realize controllable available moisture and improve fermentation yield in SSF, because it provides a theoretical basis for the optimization and improvement of fermentation modes and bioreactors. Besides, the modified SSF system could address the shortcomings of inhomogeneous culture of traditional SSF. What's more, this study can provide a technical platform for water controlling in SSF, guide the scale-up of liquid-supply SSF, accelerate the process of industrialization, modernization, mechanization, and scale-up of SSF. For example, we have employed this strategy in mold growth such as the mushroom culture, which will bring substantial economic benefits.

## Data Availability Statement

All datasets generated for this study are included in the manuscript/Supplementary Files.

## Author Contributions

Conceptualization of the study was conducted jointly by QH, MS, SH, and JQ. The methodological approach was designed and carried out by QH. Data validation and analysis were jointly carried out by QH, MS, SH, and JQ. QH prepared the original draft of the manuscript. HP participated in results and discussion, revision, and correction of the manuscript. JG participated in data analysis and revision of the manuscript. The manuscript was jointly finalized by all authors.

### Conflict of Interest

JG was employed by company Nantong Lianhai Weijing Biology Co., Ltd. The remaining authors declare that the research was conducted in the absence of any commercial or financial relationships that could be construed as a potential conflict of interest.
