# Supplementary material for: Humidity Control Strategies for Solid-State Fermentation: Capillary Water Supply by Water-Retention Materials and Negative-Pressure Auto-controlled Irrigation
Source: Front Bioeng Biotechnol. 2019 Oct 17;7:263. doi: 10.3389/fbioe.2019.00263 (PMC6812397; doi:10.3389/fbioe.2019.00263)
Supplement: Supplementary file 1 [file Table_1.docx]

**Humidity control strategies for solid-state fermentation:**

**Capillary water supply by water-retention materials and negative-pressure auto-controlled irrigation**

Qin He^1,2*^,^[[1]](#footnote-1)^Huadong Peng^2^, Mengyao Sheng^1^, Shishan Hu^1^, Jiguo Qiu^1^, Jiayu Gu^3^

Running title: Capillary water supply in solid-state fermentation

Email address:

QH: qhe@njau.edu.cn

**Table S1 Selection of model strains**

**Table S2 Program design for response surface experiment**

**Table S3 Fitting parameters of water characteristic curve of different substrates**

**Figure S1 Photos of superabsorbent polymers (SAPs) before and after water absorption**

**Figure S2 Water absorption and evaporation curve of WRMs**

a) Water absorption curve; b) Water evaporation curve.

**Figure S3 Effect of WRMs addition on MC during cellulase production**

a)-d) Cold model test for control, KL; KM and PUF, respectively; A)-D) Hot model test for control, KL; KM and PUF, respectively. The added dry WRMs amount was 0.2% of wet substrate.

**Figure S4 Response surface analysis of LS-SSF by SAPs**

**Table S1 Selection of model strains**

| Strains | Culture period (d) | Evaluation indexes |
| --- | --- | --- |
| *Trichoderma reesei* YG3 | 5-7 | Cellulase activity, biomass |
| *Trichoderma harzianum* 3.5364 | 10-12 | Amount of spores |
| *Fomes fomentarius* 5.132 | 28-30 | Laccase activity |

**Table S2 Program design for response surface experiment**

| Ru-n | Symbol code | | | *Trichoderma reesei* YG3 | | *Trichoderma harzianum* 3.5364 | | *Fomes fomentarius* 5.132 | |
| --- | --- | --- | --- | --- | --- | --- | --- | --- | --- |
|  | SAP size  (SAP-KM Percent)^a^ | Substrate size  (5 mm matrix Percent)^b^ | SAP amount  (Percent)^c^ | FPA  (U/g DW) | MC (%) | Spores number (10^9^/g DW) | MC (%) | Laccase activity  (U/g DW) | MC (%) |
| 1 | -1.68 | 0.00 | 0.00 | 4.24 | 65.59 | 20.08 | 74.32 | 1.14 | 24.39 |
| 2 | -1.00 | -1.00 | -1.00 | 4.62 | 64.97 | 23.27 | 70.43 | 2.50 | 24.63 |
| 3 | 0.00 | -1.68 | 0.00 | 4.69 | 67.26 | 13.73 | 72.30 | 2.48 | 23.79 |
| 4 | 0.00 | 0.00 | 0.00 | 4.54 | 66.74 | 8.63 | 72.60 | 1.66 | 23.97 |
| 5 | -1.00 | 1.00 | -1.00 | 4.79 | 63.82 | 32.71 | 70.78 | 1.99 | 25.07 |
| 6 | 1.00 | 1.00 | 1.00 | 3.93 | 69.84 | 23.67 | 74.24 | 2.78 | 22.91 |
| 7 | 1.00 | -1.00 | 1.00 | 4.00 | 70.22 | 14.53 | 73.41 | 1.40 | 22.79 |
| 8 | 0.00 | 1.68 | 0.00 | 4.11 | 65.75 | 14.07 | 71.50 | 1.13 | 24.33 |
| 9 | -1.00 | 1.00 | 1.00 | 2.45 | 68.82 | 13.27 | 73.63 | 3.33 | 23.25 |
| 10 | 0.00 | 0.00 | 0.00 | 4.59 | 66.23 | 14.11 | 71.42 | 1.70 | 24.16 |
| 11 | 0.00 | 0.00 | 0.00 | 4.64 | 66.01 | 14.44 | 70.48 | 1.75 | 24.24 |
| 12 | 0.00 | 0.00 | 0.00 | 4.52 | 66.52 | 15.66 | 70.07 | 1.89 | 24.05 |
| 13 | 0.00 | 0.00 | 0.00 | 4.46 | 66.19 | 15.95 | 68.32 | 1.74 | 24.17 |
| 14 | -1.00 | -1.00 | 1.00 | 3.96 | 69.45 | 13.95 | 76.98 | 2.95 | 23.04 |
| 15 | 0.00 | 0.00 | 1.68 | 2.68 | 71.02 | 13.26 | 76.65 | 2.77 | 22.53 |
| 16 | 0.00 | 0.00 | -1.68 | 5.83 | 62.60 | 16.40 | 68.71 | 4.34 | 25.56 |
| 17 | 0.00 | 0.00 | 0.00 | 4.09 | 66.71 | 12.73 | 73.50 | 1.53 | 23.99 |
| 18 | 1.00 | 1.00 | -1.00 | 5.37 | 65.53 | 10.32 | 56.22 | 3.57 | 24.42 |
| 19 | 1.68 | 0.00 | 0.00 | 4.55 | 66.87 | 16.07 | 69.38 | 2.36 | 23.93 |
| 20 | 1.00 | -1.00 | -1.00 | 4.69 | 65.43 | 12.89 | 77.87 | 1.62 | 24.45 |

^a^ SAPs size (SAP-KM Percent), its coded value of -1.68, -1, 0, 1 and 1.68 corresponded to 0, 20.24%, 50%, 70.96% and 100%, respectively.

^b^ Substrate size (5 mm matrix Percent), its coded value of -1.68, -1, 0, 1 and 1.68 corresponded to 0, 20.24%, 50%, 70.96% and 100%, respectively.

^c^ SAPs amount (Percent), its coded value of -1.68, -1, 0, 1 and 1.68 corresponded to 0.1, 0.181%, 0.3%, 0.419% and 0.5%, respectively.

**Table S3 Fitting parameters of water characteristic curve of different substrates**

| Parameters | A:  Chopped CS  (0.43 mm) | B:  Wheat bran | C:  SECS  (5 mm) | D:  Chopped SECS  (0.43 mm) |
| --- | --- | --- | --- | --- |
|  | 0.3507 | 0.2036 | 0.1015 | 0.0515 |
|  | 0.4258 | 0.3419 | 0.7611 | 0.6067 |
| *α* | 0.0271 | 0.0285 | 0.7005 | 1.9001 |
| *n* | 2.7516 | 8.6096 | 1.2526 | 1.1006 |
| *R*^2^ | 0.9997 | 0.9996 | 0.9789 | 0.9742 |


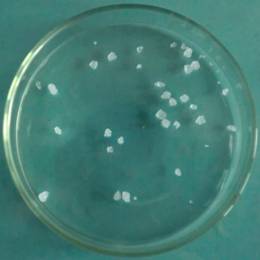

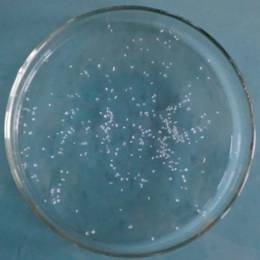


SAP-KL before water absorption SAP-KM before water absorption


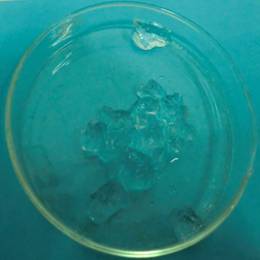

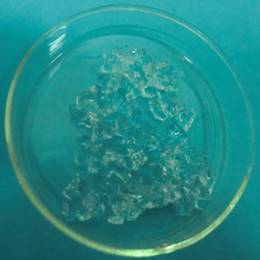


SAP-KL after water absorption SAP-KM after water absorption

**Figure S1 Photos of superabsorbent polymers (SAPs) before and after water absorption**

 **Figure S2 Water absorption and evaporation curve of WRMs**

a) Water absorption curve; b) Water evaporation curve.

**Figure S3 Effect of WRMs addition on MC during cellulase production**

a)-d) Cold model test for control, KL; KM and PUF, respectively; A)-D) Hot model test for control, KL; KM and PUF, respectively. The added dry WRMs amount was 0.2% of wet substrate.

b

c

d

a

e

f

h

g

l

j

i

k

**Figure S4 Response surface analysis of LS-SSF by SAPs**

1. ^*^Correspondence: Qin He, qhe@njau.edu.cn

   ^1^ Department of Microbiology, Key Lab of Microbiological Engineering of Agricultural Environment, Ministry of Agriculture, College of Life Sciences, Nanjing Agricultural University, Nanjing, 210095, PR China

   ^2^ Imperial College Centre for Synthetic Biology and Department of Bioengineering, Imperial College London, London, UK

   ^3^ Nantong Lianhai Weijing biology Co., Ltd, Haimen, 226133, Jiangsu, PR China  [↑](#footnote-ref-1)
